# Electronic cigarette and cannabis use: results from the 2018 Maryland Youth Risk Behavior Survey

**DOI:** 10.1186/s42238-021-00080-2

**Published:** 2021-06-25

**Authors:** Amanda Luken, Johannes Thrul, Renee M. Johnson

**Affiliations:** 1 Department of Mental Health, Johns Hopkins Bloomberg School of Public Health, 624 N Broadway, Baltimore, Maryland 21205 USA; 2 Sidney Kimmel Comprehensive Cancer Center at Johns Hopkins, Baltimore, Maryland USA; 3 Centre for Alcohol Policy Research, La Trobe University, Melbourne, Australia

**Keywords:** E-cigarettes, Cannabis, Youth Risk Behavior Survey, Complex survey sample, Adolescent, substance use

## Abstract

**Objective:**

To determine the relationship between lifetime e-cigarette use and current cannabis use among youth. Our analyses accounted for county variability, in addition to student-level covariates.

**Methods:**

This study examined responses from high school students on a state-level population survey, the 2018 Maryland Youth Risk Behavior Survey/Youth Tobacco Survey, a cross-sectional, complex survey sample. Of participating students, final analyses included an unweighted sample of 41,091 9th to 12th grade students who provided complete reports for measured variables. Analyses with survey weights were conducted between August 2019 and May 2020. A multivariable logistic regression was conducted to investigate the association between lifetime e-cigarette use and current (past 30-day) cannabis use, after controlling for county, lifetime cigarette use, current (past 30-day) alcohol use, emotional distress, and demographics.

**Results:**

Lifetime e-cigarette use significantly increased the odds of current cannabis use among Maryland high school students (aOR = 6.04; 95% CI 5.27, 6.93). Other significant risk factors for current cannabis use included lifetime cigarette use (aOR 2.23, 95% CI 1.86, 2.68) and current alcohol use (aOR 5.21, 95% CI 4.42, 6.14). Significantly higher odds of current cannabis use were also found among older high school students, males, non-Hispanic Blacks and students identifying as other race, and those reporting emotional distress.

**Conclusions:**

Lifetime e-cigarette use among Maryland high school students is strongly associated with current cannabis use when including counties as a covariate. Non-significant county differences, however, suggest smaller geographical units may be required to control for variability. Efforts should focus on reducing youth e-cigarette use to decrease cannabis use. Maryland’s recent implementation of Tobacco 21 and a ban on flavored e-cigarettes will be of interest for future evaluations.

**Supplementary Information:**

The online version contains supplementary material available at 10.1186/s42238-021-00080-2.

## Introduction

Since 2014, electronic cigarettes (e-cigarettes) have been the most commonly used tobacco product among youth, when the prevalence of their use surpassed cigarettes (Cullen et al. 2018; Gentzke et al. 2019). Youth cigarette use has been decreasing since the mid-1990s (Johnston et al. 2014) whereas estimates of the prevalence of cannabis use have remained relatively stable at roughly 20% during that same time period (CDC 2017; Johnson et al. 2015). In 2011, just 1.5% of US high school students reported past 30-day use of e-cigarettes; the prevalence increased 8- to 13-fold by 2018 (Cullen et al. 2018). This increase corresponds with the direct marketing of e-cigarettes to adolescents by e-cigarette companies, namely JUUL, beginning in 2014 (Jones and Salzman 2020).

Historically, the use of cigarettes preceded cannabis use initiation (Johnston et al. 2014; Kandel 2002), but with the decrease in cigarette use and emergence of e-cigarettes (Levy et al. 2019), youth are increasingly initiating substance use with e-cigarettes (rather than cigarettes) and are subsequently transitioning to cannabis use (Chadi et al. 2019; Dai et al. 2018; Park et al. 2020; Silveira et al. 2018; Audrain-McGovern et al. 2018). Recent meta-analyses show that e-cigarette use is associated with a greater than 3.5- to 6- fold increase in the odds of cannabis use; the magnitude of effects was stronger in studies published after 2017 (Chadi et al. 2019; Hershberger et al. 2020). Both the increasing prevalence of e-cigarette use and the increasing likelihood of transitioning from e-cigarette use to cannabis use among American youth highlight the public health importance of examining e-cigarette use as a potential risk factor for cannabis initiation.

In the present study, we examine whether a history of e-cigarette use is associated with current (i.e., past 30-day) cannabis use among a statewide sample of Maryland high school students. With differential state policies regulating access to cannabis, e-cigarettes, and cigarettes, state-level analyses can provide insight into state-specific social norms. Maryland has had an operational medical marijuana program since 2017 and a statewide decriminalization statute since 2014 (NCSL 2019). Prior to 2018, Maryland did not have any state-specific legislation regulating e-cigarettes. As elsewhere in the country, Maryland abided by the 2016 US Food and Drug Administration’s nationwide ban on the sale of e-cigarettes to youth under the age of 18. Maryland youth lifetime e-cigarette prevalence in 2017 (35.3%) was below the national average (42.2%), whereas current cannabis use (18.4%) was similar to the national prevalence of 19.8% (18.1%, 21.6%) in 2017 (CDC 2017).

## Methods

### Sample

Data came from the 2018 Maryland Youth Risk Behavior Survey/Youth Tobacco Survey (YRBS/YTS), conducted in conjunction with those two Centers for Disease Control and Prevention (CDC) surveillance systems. The Maryland dataset offers the largest YRBS sample of all the states, is designed to yield prevalence estimates at the county-level, and contains nicotine/tobacco variables from the YTS. An advantage of this analysis is the inclusion of counties as a covariate for assessing the relationship between lifetime e-cigarette use and current cannabis use to control for local youth environments. Although there are nationally representative data supporting the temporality of e-cigarette use and subsequent cannabis use (Dai et al. 2018), such studies are prone to aggregation bias (Soobader et al. 2001). By accounting for county variability, the likelihood of aggregation bias is reduced (Soobader et al. 2001). Because we explore lifetime e-cigarette use and current cannabis use, findings will enhance what is known about the possibility of e-cigarette use preceding cannabis use within the state of Maryland.

### Survey design

The YRBS is a biennial nationwide, school-based survey that monitors the prevalence of health risk behaviors as part of the CDC Youth Risk Behavior Surveillance System (Kann et al. 2018). The Youth Tobacco Survey (YTS) is also conducted by the CDC but was developed for the sole purpose of examining youth tobacco trends (CDC Youth Tobacco Survey (YTS)). The 2018 MD YRBS/YTS is a subset of the nationwide survey combined with the Youth Tobacco Survey (Maryland Department of Health a). For each county-level public school system, the MD YRBS/YTS implemented a two-stage cluster design with first stage sampling on the school-level and second-stage sampling of classrooms within schools (Kann et al. 2018). Data are representative for the state as a whole and for each county. Sample weights were developed to account for the complex sample design.

### Data collection

Items included questions from the core YRBS and YTS instruments, which address key adolescent health and risk behaviors, such as substance use, violence, nicotine/tobacco use, and demographic factors. Selected classes had students voluntarily complete the survey anonymously via pen and paper. Overall response rates for the state and each of the 24 counties exceeded 60%. The state-level YRBS study design has been published for further reference (Kann et al. 2018, Maryland Department of Health a, CDC). Secondary data analyses were exempt from IRB-approval.

### Measures

The goal of the present study was to examine the association between past 30-day cannabis use and lifetime e-cigarette use. The primary outcome was any past 30-day cannabis use. Our main exposure was a history of lifetime e-cigarette use. Our covariates included lifetime cigarette use and past 30-day alcohol use.

Current cannabis use was assessed with one question: “During the past 30 days, how many times did you use marijuana?” Marijuana was defined to students as pot, weed, or cannabis. There were six response options that ranged from 1 (signifying never) to 6 (40 or more times). The variable was dichotomized (i.e., no versus any current use).

Lifetime e-cigarette use was assessed with the question “Have you ever used an electronic vapor product?” (response options: yes/no). Electronic vapor products were defined as e-cigarettes, e-cigars, vapes, vape pens, e-hookahs, mods, and hookah pens. Students were also prompted, prior to recording their response, to consider electronic vapor products by their marketing name (i.e., JUUL, Vuse, MarkTen, and blu).

Because both cigarette and alcohol use are associated with both e-cigarette and cannabis use (Park et al. 2020; Hershberger et al. 2020; D'Amico et al. 2020; Moss et al. 2014), both were included as control variables. For age at first cigarette use, students responded to “How old were you when you smoked a whole cigarette for the first time?” To reflect lifetime cigarette use, we dichotomized responses into never and all other responses as ever. Current alcohol use was assessed with the question, “During the past 30 days, on how many days did you have at least one drink of alcohol?” Alcohol was specified to include “beer, wine, wine coolers, and liquor such as rum, gin, vodka, or whiskey” and to exclude sips taken for religious ceremonies. Responses were dichotomized into current use versus no current use.

Emotional distress was assessed with the item: “During the past 12 months, did you ever feel so sad or hopeless almost every day for two weeks or more in a row that you stopped doing some usual activities?” (response options: yes/no). This study also controlled for sex (girl or boy), grade in school (9th, 10th, 11th, 12th), and race/ethnicity (monoracial White, Black, and Asian; Hispanic/Latinx, any race; and non-Hispanic, all other racial groups). The “other” category includes students who selected multiple racial groups, and those who identified as American Indian/Alaska Native or Native Hawaiian/Pacific Islander.

### Data analyses

We conducted Rao-Scott χ^2^ tests to assess the association between current cannabis use and all variables of interest. In the main analysis, we used a multivariable logistic regression model to assess the association between current cannabis use (outcome) and lifetime e-cigarette use (exposure), adjusting for lifetime cigarette use, current alcohol use, sex, grade in school, race/ethnicity, county, and emotional distress. Counties were coded via effect coding, specifically deviant coding, where the mean for each county was compared to the overall mean (UCLA: Statistical Consulting Group 2011). We derived adjusted odds ratios (aORs) and 95% confidence intervals (CIs) from the multivariable logistic regression models. There was a possibility that many of the students reporting lifetime e-cigarette use or cigarette use were also currently using e-cigarettes or cigarettes. To identify if the same findings held for those who previously used e-cigarettes or cigarettes but were not necessarily still using e-cigarettes or cigarettes, we conducted a sensitivity analysis by removing current e-cigarette and cigarette users from the model. Complex survey analyses were conducted in R version 4.0.0 via the *survey* package, with sample weights applied (Lumley 2004). All analyses and reported values reflected weighted results. Statistical significance was indicated for *p* < 0.05.

## Results

The 2018 Maryland YRBS/YTS sample included 41,091 students (weighted *n* = 257,086). Students missing data on key study variables (5,646 students) were excluded from the analytical sample (Supplemental Table 1), resulting in a final analytical sample size of 201,282. Students excluded from analyses were significantly different from included students on all measures. For example, excluded students were more likely to identify as male and Black or Hispanic and were more likely to report lifetime e-cigarette use, lifetime cigarette use, current alcohol use, and current cannabis use, compared to included students (Supplemental Table 2). Forty-two percent were White, 31% were Black, and 15% were Hispanic/Latinx (Table 1). More than one third (35.4%) of all students reported lifetime e-cigarette use, 6.9% reported lifetime cigarette use, and 22.5% reported current alcohol use. Thirty-one percent reported emotional distress.

**Table 1:** Weighted sample characteristics among Maryland High School Students, 2018 Maryland Youth Risk Behavior Survey

	Total		Current cannabis use			
			No		Yes	
	N = 201,282		N = 168,909		N = 32,373	
	N	Weighted % (95% CI)	N	Weighted % (95% CI)	N	Weighted % (95% CI)
**Sex**						
Girls	102,631	51.0 (49.5–52.5)	85,064	50.4 (48.8–51.9)	17,567	54.3 (52.0–56.5)
Boys	98,651	49.0 (47.5–50.5)	83,845	49.6 (48.1–51.2)	14,806	45.7 (43.5–48.0)
**Grade**						
9th	54,719	27.2 (24.3–30.3)	49,662	29.4 (26.3–32.7)	5057	15.6 (13.2–18.3)
10th	52,473	26.1 (23.5–28.8)	44,986	26.6 (23.9–29.5)	7487	23.1 (20.5–25.9)
11th	47,543	23.6 (21.5–25.9)	38,892	23.0 (20.8–25.4)	8651	26.7 (24.1–29.6)
12th	46,547	23.1 (21.0–25.4)	35,369	20.9 (18.9–23.1)	11,177	34.5 (31.3–37.9)
**Race/Ethnicity**						
Non-Hispanic White	85,225	42.3 (40.5–44.2)	70,100	41.5 (39.6–43.5)	15,125	46.7 (43.7–49.8)
Non-Hispanic Black	62,228	30.9 (28.7–33.2)	52,160	30.9 (28.7–33.2)	10,067	31.1 (27.9–34.5)
Hispanic/Latinx, any race	29,437	14.6 (13.0–16.5)	25,243	14.9 (13.0–17.1)	4193	13.0 (11.7–14.4)
Non-Hispanic Asian	13,873	6.9 (5.6–8.4)	12,948	7.7 (6.2–9.4)	924	2.9 (2.0–4.0)
Non-Hispanic, all other races	10,520	5.2 (4.9–5.6)	8457	5.0 (4.7–5.3)	2063	6.4 (5.5–7.3)
**Lifetime e-cigarette use**						
Yes	71,259	35.4 (34.2–36.7)	45,376	26.9 (25.8–28.0)	25,883	80.0 (77.6–82.1)
No	130,024	64.6 (63.3–65.8)	123,533	73.1 (72.0–74.2)	6490	20.0 (17.9–22.4)
**Lifetime cigarette use**						
Yes	13,830	6.9 (6.3–7.5)	25,064	3.9 (3.3–4.5)	7309	22.6 (20.8–24.5)
No	187,452	93.1 (92.5–93.7)	162,388	96.1 (95.5 – 96.7)	25,064	77.4 (75.5–79.2)
**Current alcohol use**						
Yes	45,194	22.5 (21.2–23.7)	24,537	14.5 (13.6–15.5)	20,657	63.8 (60.9–66.6)
No	156,088	77.5 (76.3–78.8)	144,372	85.5 (84.5–86.4)	11,716	36.2 (33.4–39.1)
**Emotional distress**						
Yes	61,924	30.8 (29.7–31.8)	46,741	27.7 (26.7–28.7)	15,183	46.9 (44.4–49.4)
No	139,358	69.2 (68.2–70.3)	122,168	72.3 (71.3–73.3)	17,190	53.1 (50.6–55.6)

Note: Excluding students missing responses to current cannabis use or covariates and those who reported ungraded. Data are rounded

Sums within groups may slightly differ from sample size due to rounding weights

All chi-squared results were statistically significant (p < .001)

Sixteen percent of Maryland high school students reported current cannabis use. Students who reported current cannabis use were more often girls (54.3%), higher grade levels (9th, 15.6%; 10th, 23.1%; 11th, 26.7%; 12th, 34.5%) and identified as White (46.7%) or Black (31.1%). There were statistically significant differences across all variables for students reporting current cannabis use versus students who reported no current cannabis use, and especially more pronounced differences in lifetime cigarette use (22.6% vs. 3.9%), current alcohol use (63.8% vs. 14.5%), and emotional distress (46.9% vs. 27.7%) for current cannabis use versus no current cannabis use, respectively.

We used logistic regression to determine the association between lifetime e-cigarette use and current cannabis use, adjusting for lifetime cigarette use, current alcohol use, emotional distress, race/ethnicity, sex, grade in school, and county. The odds of current cannabis use was 6.04 (95% CI 5.27, 6.93) among those reporting any (versus no) lifetime e-cigarette use (Table 2). Lifetime cigarette use and current alcohol use were also strongly associated with current cannabis use; respectively, adjusted ORs were 2.23 (95% CI 1.86, 2.68) and 5.21 (95% CI 4.42, 6.14). Odds of current cannabis were higher among 10th, 11th, and 12th graders relative to 9th graders, as well as among those reporting emotional distress. The odds of current cannabis use were higher among non-Hispanic Black students and those in the non-Hispanic Other category, relative to non-Hispanic Whites, and were lower among non-Hispanic Asian students. The final multivariable model also controlled for Maryland counties as additional covariate. Only a few counties demonstrated significant differences in the odds of youth current cannabis use from the overall mean across all counties (results not shown). In the sensitivity analysis, we conducted a logistic regression without students who indicated that they used an e-cigarette or cigarette within the past 30-days and found the same associations, except with regards to lifetime cigarette use which was no longer significantly associated with current cannabis use. We also conducted chi-squared analyses for included versus excluded students and found that excluded students were more likely to confirm lifetime e-cigarette use and current cannabis use.

**Table 2:** Risk for current cannabis use

Variables	Adjusted odds ratio (95% CI)
**Lifetime e-cigarette use**	
No	Reference
Yes	6.04 (5.27, 6.93)
**Lifetime cigarette use**	
No	Reference
Yes	2.23 (1.86, 2.68)
**Current alcohol use**	
No	Reference
Yes	5.21 (4.42, 6.14)
**Sex**	
Girls	Reference
Boys	1.04 (0.93, 1.16)
**Grade**	
9th grade	Reference
10th grade	1.30 (1.05, 1.59)
11th grade	1.57 (1.33, 1.85)
12th grade	1.91 (1.58, 2.31)
**Race/ethnicity**	
Non-Hispanic White	Reference
Non-Hispanic Black	1.99 (1.67, 2.37)
Hispanic/Latinx	1.14 (0.91, 1.42)
Non-Hispanic Asian	0.60 (0.36, 0.97)
Non-Hispanic other	1.66 (1.37, 2.02)
**Emotional distress**	
No	Reference
Yes	1.40 (1.23, 1.59)

Note: A multivariable logistic regression controlling for all variables including counties

## Discussion

The purpose of our study was to characterize the association between current cannabis use and lifetime e-cigarette use. Our results showed a cross-sectional association between lifetime e-cigarette use and current cannabis use, even after adjustment for lifetime cigarette use, current alcohol use, emotional distress, and demographic factors. This finding provides additional evidence that lifetime e-cigarette use may be associated with current cannabis use among youth, concordant with the findings from the PATH study (Population Assessment of Tobacco and Health) — a longitudinal nationwide cohort of people aged 12 and up (Dai et al. 2018). The PATH study showed that youth with lifetime e-cigarette use had an increased likelihood of initiating cannabis a year later, compared to never e-cigarette users (Dai et al. 2018). Our findings build upon existing research on the relationship between lifetime e-cigarette use and current cannabis use (Dai et al. 2018; Chadi et al. 2019; Park et al. 2020; Silveira et al. 2018; Audrain-McGovern et al. 2018) by providing evidence with a large statewide representative sample of high school student in Maryland and by accounting for other substance use and county differences.

### Explanations for the e-cigarette-cannabis association

Lifetime e-cigarette use was associated with the greatest odds of current cannabis use compared to all other variables, including lifetime cigarette use. The relationship between e-cigarette use and subsequent cannabis use among youth may be due to several reasons. First, lifetime e-cigarette use, regardless of frequency of use, has been shown to increase the risk of future substance use (Park et al. 2020; Silveira et al. 2018), potentially due to nicotine’s activation of the developing brain’s reward and pleasure pathways (Yuan et al. 2015). Even in low doses, nicotine may alter neural pathways that persist into adulthood (Yuan et al. 2015). Second, peer and adult substance use influence youth’s substance use decisions (Thrul et al. 2014). Through their social circles, youth may model the health risk behaviors of peers and adults (Cassidy et al. 2018), including the perceived behaviors of individuals in their social network (Thrul et al. 2014). Additionally, there is an increasing pattern of concurrent cannabis and nicotine use in e-cigarette devices (Park et al. 2020; Nicksic et al. 2020; Tucker et al. 2019; Trivers et al. 2018). E-liquids or concentrates containing tetrahydrocannabinol (THC) can be administered in e-cigarette or vaporizer devices, which may be a more convenient and discrete form of cannabis use (Jones et al. 2016). Co-administration of cannabis and nicotine within e-cigarette devices has been found to increase from 8th grade (2.6%) to 12th grade (8.5%) (Dai and Hao 2016). Thus, the current findings may also reflect co-administration of cannabis and nicotine in e-cigarette devices. Alternatively, the association between e-cigarette use and cannabis use among Maryland adolescents could simply reflect a predisposition to using cannabis, regardless of whether e-cigarettes were used (Morral et al., 2002).

### Associations with socio-demographic factors

Higher grade levels, greater emotional distress, and non-Hispanic Black and non-Hispanic other racial identities were associated with greater odds of current cannabis use compared to lower grade levels and non-Hispanic White students. Greater odds of cannabis use at higher grade levels is in line with existing literature, in which cannabis use has been found to be the highest among 18–25 year olds (Substance Abuse and Mental Health Services Administration 2019). Students experiencing emotional distress may have greater odds of cannabis use, because they are using it as a coping strategy (Khantzian 1997). The elevated odds of current cannabis use among students identifying as Non-Hispanic Black may be due to the popularity of blunts (Golub et al. 2006). The non-Hispanic other group comprised students identifying as American Indian/Alaska Native or multiracial students, among others, of which both have also been shown to have higher cannabis use in the literature (Johnson et al. 2015; Keyes et al. 2017). Although more research needs to be done to better understand the individual and structural-level contributors of these racial disparities in adolescent cannabis use, racial discrimination, violence towards and within these communities, and the associated stress of one’s racial minority status coupled with the experiences of daily life may lead to substance use for the alleviation of emotional distress (Clark et al. 2015; Khantzian 1997). Nevertheless, these findings underline the need for public health interventions to mitigate the possible harmful effects of these racial disparities in cannabis use among high school students.

### Implications for Maryland and the USA

Despite the high likelihood for nicotine dependence following e-cigarette use, youth are unlikely to view use as risky (Amrock et al. 2016; Chen et al. 2020). It remains critical to prevent adolescent use of e-cigarettes. In response to the number of vaping-related deaths in the USA in fall 2019, the state of Maryland in 2020 expanded the national ban on the sale of certain e-cigarette flavors to encompass all flavorings, except for tobacco and menthol (Kramer et al. 2020). Maryland is the first state to expand the ban to almost all flavorings, which can reduce the appeal of e-cigarettes for youth. Additionally, in 2019, Maryland increased the legal age from 18 to 21 for the purchase of tobacco and nicotine products (Tobacco 21) (Maryland Department of Health b), as many other states have done (Preventing Tobacco Addiction Foundation). As a result, future studies may benefit from examining the effect of both the flavoring ban and Tobacco 21 on e-cigarette, cannabis, and alcohol use among youth in Maryland to inform policy makers in other states and federally. Despite these policy changes, it may prove difficult to enforce these laws in online markets, and early education about the harms of e-cigarette and cannabis use for youth is needed in addition to tobacco control policy measures.

The economic principle of complementary goods suggests that when one commonly used substance is made more difficult to access, there are decreases in other substances that are used concurrently, leading to lower overall consumption. Therefore, efforts to limit youth access to e-cigarette may also prevent cannabis use. Additionally, to the extent that our findings reflect a sequential process of initiation of e-cigarettes and then cannabis, ongoing efforts to prevent adolescent e-cigarette use could have a ripple effect of preventing initiation of cannabis use. Additional studies will be needed to test these possibilities.

### Limitations

This study had a number of limitations. First, we estimated lifetime e-cigarette use and current cannabis use as binary variables, which may result in the inclusion of students who tried e-cigarettes or cannabis once. Further, due to the cross-sectional nature of the study and the study questions, themselves, we could not establish temporality or causality; however, the present study found the same associations as other studies with the means for temporality in a state-specific sample of adolescents while accounting for county-level variation. Second, there were significant differences between excluded and included participants across all variables. Our results were sensitive enough to detect a strong association between lifetime e-cigarette use and current cannabis use without those students excluded, who were more likely to have reported lifetime e-cigarette use or current cannabis use. Non-response bias from voluntary surveys, such as the MD YRBS/YTS, tends to result in more positive health outcomes reported among survey respondents (Cheung et al. 2017). As a result, our findings may underestimate the true relationship between lifetime e-cigarette use and current cannabis use. It is also possible that our findings may overestimate the relationship between lifetime e-cigarette use and current cannabis use due to the prevalence of current cannabis use exceeding 10% (Davies et al. 1998).

## Conclusion

We found that lifetime e-cigarette use was associated with increased odds of current cannabis use among Maryland youth, suggesting that successful e-cigarette prevention programs may affect youth cannabis use. In addition to Maryland’s implementation of Tobacco 21, which aims to decrease access to tobacco products for youth, prevention programs can complement the decrease in availability of tobacco products with campaigns in both physical and online environments that decrease youth’s desire for seeking out e-cigarettes. While Tobacco 21 may decrease physical, local access, much of future efforts to prevent e-cigarette and cannabis use may require increasing smoking cessation among older peers and adults who may continue to model substance use among youth.

## Supplementary Information

**Additional file 1:.** Supplemental Table 1. Unweighted Sample Characteristics Missing among Maryland High School Students, 2018 Maryland Youth Risk Behavior Survey. Supplemental Table 2. Unweighted Differences in Included and Excluded Maryland High School Students, 2018 Maryland Youth Risk Behavior Survey

## Data Availability

To request data, send a request by email to mdh.yrbs_ytsdatarequest1@maryland.gov indicating a description of what data is being requested and what the data will be used for.

## Abbreviations

aOR: Adjusted odds ratio
CDC: Center for Disease Control and Prevention
CI: Confidence interval
e-cigarette: Electronic cigarette
MD: Maryland
PATH: Population Assessment of Tobacco and Health
THC: Tetrahydrocannabinol
YRBS/YTS: Youth Risk Behavior Survey/Youth Tobacco Survey

## References

[CR1] Amrock SM, Lee L, Weitzman M (2016). Perceptions of e-cigarettes and noncigarette tobacco products among US youth. Pediatrics..

[CR2] Audrain-McGovern J, Stone MD, Barrington-Trimis J, Unger JB, Leventhal AM (2018). Adolescent e-cigarette, hookah, and conventional cigarette use and subsequent marijuana use. Pediatrics..

[CR3] Cassidy RN, Meisel MK, DiGuiseppi G, Balestrieri S, Barnett NP (2018). Initiation of vaporizing cannabis: Individual and social network predictors in a longitudinal study of young adults. Drug Alcohol Depend..

[CR4] CDC (2017) High School YRBS. https://nccd.cdc.gov/youthonline/App/Default.aspx. Accessed April 10, 2020.

[CR5] CDC Youth Tobacco Survey (YTS). https://www.cdc.gov/tobacco/data_statistics/surveys/yts/index.htm. Accessed August 3, 2020.

[CR6] Chadi N, Schroeder R, Jensen JW, Levy S (2019). Association between electronic cigarette use and marijuana use among adolescents and young adults: a systematic review and meta-analysis. JAMA Pediatr..

[CR7] Chen Y, Tilden C, Vernberg DK (2020). Adolescents’ interpretations of e-cigarette advertising and their engagement with e-cigarette information: results from five focus groups. Psychol Health..

[CR8] Cheung KL, ten Klooster PM, Smit C, de Vries H, Pieterse ME (2017). The impact of non-response bias due to sampling in public health studies: a comparison of voluntary versus mandatory recruitment in a Dutch national survey on adolescent health. BMC Public Health..

[CR9] Clark TT, Salas-Wright CP, Vaughn MG, Whitfield KE (2015). Everyday discrimination and mood and substance use disorders: a latent profile analysis with African Americans and Caribbean Blacks. Addict Behav..

[CR10] Cullen KA, Ambrose BK, Gentzke AS, Apelberg BJ, Jamal A, King BA (2018). Notes from the field: use of electronic cigarettes and any tobacco product among middle and high school students — United States, 2011–2018. MMWR Morb Mortal Wkly Rep..

[CR11] Dai H, Hao J (2016). Electronic cigarette and marijuana use among youth in the United States. Addict Behav..

[CR12] Dai H, Catley D, Richter KP, Goggin K, Ellerbeck EF (2018). Electronic cigarettes and future marijuana use: a longitudinal study. Pediatrics..

[CR13] D'Amico EJ, Rodriguez A, Tucker JS, Dunbar MS, Pedersen ER, Shih RA, Davis JP, Seelam R (2020). Early and late adolescent factors that predict co-use of cannabis with alcohol and tobacco in young adulthood. Prevention science: the official journal of the Society for Prevention Research..

[CR14] Davies HTO, Crombie IK, Tavakoli M (1998). When can odds ratios mislead?. BMJ..

[CR15] Gentzke AS, Creamer M, Cullen KA, Ambrose BK, Willis G, Jamal A, King BA (2019). Vital signs: tobacco product use among middle and high school students - United States, 2011-2018. MMWR Morb Mortal Wkly Rep..

[CR16] Golub A, Johnson BD, Dunlap E (2006). The growth in marijuana use among American youths during the 1990s and the extent of blunt smoking. Journal of Ethnicity in Substance Abuse..

[CR17] Hershberger A, Argyriou E, Cyders M (2020). Electronic nicotine delivery system use is related to higher odds of alcohol and marijuana use in adolescents: meta-analytic evidence. Addictive Behaviors..

[CR18] Johnson RM, Fairman B, Gilreath T, Xuan Z, Rothman EF, Parnham T, Furr-Holden DM (2015). Past 15-year trends in adolescent marijuana use: differences by race/ethnicity and sex. Drug Alcohol Depend..

[CR19] Johnston LD, O'Malley PM, Miech RA, Bachman JG, Schulenberg JE. Monitoring the future national results on drug use: 1975 - 2013: overview, key findings on adolescent drug use: Institute for Social Research, The University of Michigan; 2014.

[CR20] Jones K, Salzman GA (2020). The vaping epidemic in adolescents. Mo Med..

[CR21] Jones CB, Hill ML, Pardini DA, Meier MH (2016). Prevalence and correlates of vaping cannabis in a sample of young adults. Psychol Addict Behav..

[CR22] Kandel D (2002). Stages and pathways of drug involvement: examining the gateway hypothesis.

[CR23] Kann L, McManus T, Harris WA, Shanklin SL, Flint KH, Queen B, Lowry R, Chyen D, Whittle L, Thornton J, Lim C, Bradford D, Yamakawa Y, Leon M, Brener N, Ethier KA (2018). Youth Risk Behavior Surveillance — United States, 2017. MMWR Surveill Summ..

[CR24] Keyes KM, Wall M, Feng T, Cerda M, Hasin D (2017). Race/ethnicity and marijuana use in the United States: diminishing differences in the prevalence of use, 2006 to 2015. Drug Alcohol Depend..

[CR25] Khantzian EJ (1997). The self-medication hypothesis of substance use disorders: a reconsideration and recent applications. Harv Rev Psychiatry..

[CR26] Kramer B, Rosapepe J, West C (2020). Electronic smoking devices – Flavor prohibition.

[CR27] Levy DT, Warner KE, Cummings KM, Hammond D, Kuo C, Fong GT, Thrasher JF, Lukasz Goniewicz M, Borland R (2019). Examining the relationship of vaping to smoking initiation among US youth and young adults: a reality check. Tob Control..

[CR28] Lumley T (2004). Analysis of complex survey samples. J Stat Softw..

[CR29] Maryland Department of Health (a) The Maryland Youth Risk Behavior Survey & Youth Tobacco Survey - YRBS/YTS. Maryland Department of Health, Prevention and Health Promotion Administration. https://phpa.health.maryland.gov/ccdpc/Reports/Pages/YRBS-Main.aspx. Accessed April 1, 2020.

[CR30] Maryland Department of Health (b) Tobacco 21 FAQ. Maryland Department of Health, Prevention and Health Promotion. https://health.maryland.gov/notobaccosalestominors/Pages/Tobacco%2021%20FAQ.aspx. Accessed August 3, 2020.

[CR31] Morral AR, McCaffrey DF, Paddock SM (2002). Reassessing the marijuana gateway effect. Addiction..

[CR32] Moss HB, Chen CM, Yi H (2014). Early adolescent patterns of alcohol, cigarettes, and marijuana polysubstance use and young adult substance use outcomes in a nationally representative sample. Drug and Alcohol Dependence..

[CR33] NCSL. National Conference on State Legislators, marijuana deep dive; 2019. http://www.ncsl.org/bookstore/state-legislatures-magazine/marijuana-deep-dive.aspx.

[CR34] Nicksic NE, Do EK, Barnes AJ (2020). Cannabis legalization, tobacco prevention policies, and cannabis use in e-cigarettes among youth. Drug Alcohol Depend..

[CR35] Park E, Livingston JA, Wang W, Kwon M, Eiden RD, Chang Y (2020). Adolescent e-cigarette use trajectories and subsequent alcohol and marijuana use. Addict Behav..

[CR36] Preventing Tobacco Addiction Foundation Tobacco 21 - Preventing Tobacco Addiction Foundation. Tobacco 21. http://tobacco21.org/. Accessed August 3, 2020.

[CR37] Silveira ML, Conway KP, Green VR, Kasza KA, Sargent JD, Borek N, Stanton CA, Cohn A, Hilmi N, Cummings MK, Niaura RS, Lambert EY, Brunette MF, Zandberg I, Tanski SE, Reissig CJ, Callahan-Lyon P, Slavit WI, Hyland AJ, Compton WM (2018). Longitudinal associations between youth tobacco and substance use in waves 1 and 2 of the Population Assessment of Tobacco and Health (PATH) Study. Drug Alcohol Depend..

[CR38] Soobader M, LeClere FB, Hadden W, Maury B (2001). Using aggregate geographic data to proxy individual socioeconomic status: does size matter?. Am J Public Health..

[CR39] Substance Abuse and Mental Health Services Administration (2019). Results from the 2018 National Survey on Drug Use and Health: detailed tables.

[CR40] Thrul J, Lipperman-Kreda S, Grube J, Friend KB (2014). Community-level adult daily smoking prevalence moderates the association between adolescents’ cigarette smoking and perceived smoking by friends. J Youth Adolescence..

[CR41] Trivers KF, Phillips E, Gentzke AS, Tynan MA, Neff LJ (2018). Prevalence of cannabis use in electronic cigarettes among US youth. JAMA Pediatr..

[CR42] Tucker JS, Rodriguez A, Dunbar MS, Pedersen ER, Davis JP, Shih RA, et al. Cannabis and tobacco use and co-use: trajectories and correlates from early adolescence to emerging adulthood. Drug Alcohol Depend. 2019, 204:107499 10.1016/j.drugalcdep.2019.06.004. 10.1016/j.drugalcdep.2019.06.004 PMC6878180 31479864

[CR43] UCLA: Statistical Consulting Group. R Library Contrast Coding Systems for categorical variables. UCLA: Statistical consulting group; 2011. https://stats.idre.ucla.edu/r/library/r-library-contrast-coding-systems-for-categorical-variables/#DEVIATION. Accessed Oct 23, 2020.

[CR44] Yuan M, Cross SJ, Loughlin SE, Leslie FM (2015). Nicotine and the adolescent brain. J Physiol..

