# Supplementary material for: Electronic cigarette and cannabis use: results from the 2018 Maryland Youth Risk Behavior Survey
Source: J Cannabis Res. 2021 Jun 25;3:21. doi: 10.1186/s42238-021-00080-2 (PMC8234632; doi:10.1186/s42238-021-00080-2)
Supplement: Supplementary file 1 — Additional file 1:. Supplemental Table 1. Unweighted Sample Characteristics Missing among Maryland High School Students, 2018 Maryland Youth Risk Behavior Survey. Supplemental Table 2. Unweighted Differences in Included and Excluded Maryland High School Students, 2018 Maryland Youth Risk Behavior Survey [file 42238_2021_80_MOESM1_ESM.docx]

**Supplemental Table 1.** Unweighted Sample Characteristics Missing among Maryland High School Students, 2018 Maryland Youth Risk Behavior Survey

|  | **Missingness** | |
| --- | --- | --- |
|  | Unweighted n | % of total unweighted sample |
| **Sex** | 453 | 1.1 |
| **Grade** | 432 | 1.1 |
| **Race/Ethnicity** | 1432 | 3.5 |
| **Current cannabis use** | 2068 | 5.0 |
| **Lifetime e-cigarette use** | 1822 | 4.4 |
| **Lifetime cigarette use** | 1182 | 2.9 |
| **Current alcohol use** | 2371 | 5.8 |
| **Emotional distress** | 741 | 1.8 |
| **County** | 0 | 0.0 |

Note: Students who reported ungraded are not included in the missing variable count for grade. Data are rounded.

**Supplemental Table 2.** Unweighted Differences in Included and Excluded Maryland High School Students, 2018 Maryland Youth Risk Behavior Survey

|  | **Included** | | **Excluded^a^** | |
| --- | --- | --- | --- | --- |
|  | **N= 35445** | | **N=5646** | |
| **Sex** | N | Unweighted % | N | Unweighted % |
| Girls | 18488 | 52.2 | 2122 | 40.9 |
| Boys | 16957 | 47.8 | 3071 | 59.1 |
| **Grade** |  |  |  |  |
| 9^th^ | 10155 | 28.7 | 1553 | 29.8 |
| 10^th^ | 9878 | 27.9 | 1334 | 25.6 |
| 11^th^ | 8670 | 24.5 | 1171 | 22.5 |
| 12^th^ | 6742 | 19.0 | 989 | 19.0 |
| Ungraded | --- | --- | 167 | 3.2 |
| **Race/Ethnicity** |  |  |  |  |
| Non-Hispanic White | 19977 | 56.4 | 1523 | 36.1 |
| Non-Hispanic Black | 6726 | 19.0 | 1199 | 28.5 |
| Hispanic/Latinx, any race | 4182 | 11.8 | 893 | 21.2 |
| Non-Hispanic Asian | 1532 | 4.3 | 158 | 3.7 |
| Non-Hispanic, all Other races | 3028 | 8.5 | 441 | 10.5 |
| **Lifetime e-cigarette use** |  |  |  |  |
| Yes | 15567 | 43.9 | 2160 | 56.5 |
| No | 19878 | 56.1 | 1664 | 43.5 |
| **Lifetime cigarette use** |  |  |  |  |
| Yes | 3342 | 9.4 | 1218 | 27.3 |
| No | 32103 | 90.6 | 3246 | 72.7 |
| **Current alcohol use** |  |  |  |  |
| Yes | 9457 | 26.7 | 1202 | 36.7 |
| No | 25988 | 73.3 | 2073 | 63.3 |
| **Emotional distress** |  |  |  |  |
| Yes | 11224 | 31.7 | 1790 | 36.5 |
| No | 24221 | 68.3 | 3115 | 63.5 |
| **Current cannabis use** |  |  |  |  |
| Yes | 6067 | 17.4 | 1128 | 27.4 |
| No | 28846 | 82.6 | 2982 | 72.6 |

^a^Excluded students were missing data on one of the variables in the model and/or reported ungraded as a grade. Subgroups may not equal the total due to missingness.
